# Kaposi Sarcoma in an Immunocompetent Patient Treated With Paclitaxel

**DOI:** 10.7759/cureus.33641

**Published:** 2023-01-11

**Authors:** Sunakshi Sharma, Vikas Malgotra, Mashkoor Wani

**Affiliations:** 1 Dermatology, Government Medical College, Jammu, IND; 2 Dermatology, Rama Medical College, Hapur, IND

**Keywords:** kaposi sarcoma in an immunocompetent patient, kaposi sarcoma good immunity, kaposi sarcoma paclitaxel, kaposi sarcoma hiv negative, kaposi sarcoma

## Abstract

Kaposi sarcoma (KS) is a rare multifocal tumor originating from the cells lining the blood vessels. It is characterized by vascular proliferation and is usually associated with immunosuppression due to multiple factors, including AIDS or organ transplantation. However, sporadic cases of KS have been reported in HIV-negative immunocompetent patients. Paclitaxel has shown efficacy and may be an alternative for the initial therapy of patients with KS. We report a case of KS in a 70-year-old Asian male who was HIV-negative, with no history of immunosuppression or homosexuality and a good response to treatment with Paclitaxel.

## Introduction

Kaposi sarcoma (KS) is a rare multifocal tumor associated with human herpes virus 8 (HHV-8) infection. It is characterized by purplish, red-blue, or brown-black macules, papules, and nodules prone to bleeding and ulceration. It was first described in 1872 by Moritz Kaposi, an Austro-Hungarian dermatologist, who termed it *idiopathic multiple-pigmented sarcoma of skin*. It is usually associated with HIV infection, immunodeficiency, or immunosuppressive therapy. KS is 20,000-fold more common in AIDS patients who have not had highly active antiretroviral therapy (HAART) [1]. Infection with HHV-8 is associated with multiple cofactors, which give rise to the tumor. KS has four distinct subtypes, i.e., classic, endemic, iatrogenic, and HIV-associated. Its natural history is heterogeneous and varies from indolent to more aggressive and fatal in the anaplastic variants.

## Case presentation

A 72-year-old male patient presented to a tertiary care hospital with a history of pink-to-purple, painless, non-itchy papules around the ankle for the last seven months, which progressively increased in size and number to involve the dorsum and sole of the right foot over four months. The lesions coalesced to form plaques and nodules of varying sizes, accompanied by edema over the next three months. He consulted many dermatologists and was treated with topical steroids. After taking multiple medications, the patient reported discharge of pus and pain over the lesions, due to which he could not walk properly. He had been receiving treatment for coronary artery disease for the last 10 years and was not affected by any other chronic illness.

**Figure 1:**
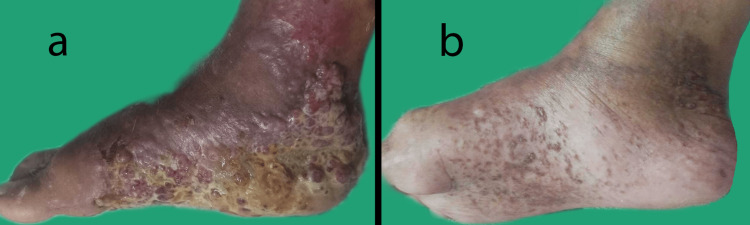
Improvement of lesions over the right foot after treatment. (a) Erythematous plaques and nodules extending over the ankle, dorsum, and sole. Yellowish exudate and discolored skin can also be seen surrounding the lesions. (b) Significant clearance of lesions after treatment with Paclitaxel.

The patient was hemodynamically stable on general examination with no regional or generalized lymphadenopathy. On local examination, multiple pinkish/violaceous papules and nodules were seen over the right foot (Figure 1a). The lesions were tender and soft to firm in consistency, with pus discharge from the lesions over the sole and edema. There were no lesions in the mouth or genitalia. The fingernails were normal, while the nails of both feet showed subungual hyperkeratosis, yellowish discoloration, and onycholysis.

Routine blood investigations and imaging studies did not reveal any significant findings. HIV serology was negative. An excisional biopsy of the lesion revealed proliferating and irregularly dilated vascular channels and spindle cells dissecting the dermal collagen and surrounding the cutaneous adnexal structures with focal slit-like spaces containing RBCs.

Immunohistochemistry markers were positive for CD31, CD34, vimentin, and HHV-8, with an immunoreactive score of 3+ for CD31 and 4+ for CD34, vimentin, and HHV-8 each. The morphological and immunohistochemistry features were consistent with plaque-stage KS. A PET scan revealed metabolically active circumferential cutaneous thickening and subcutaneous stranding involving the right lower leg, dorsal and plantar aspect of the sole of the right foot with no evidence of metabolic lesions elsewhere in the body. The patient received chemotherapy with six sittings of paclitaxel injections at two weeks intervals, with significant improvement in the lesions (Figure 1b).

## Discussion

KS is a multifocal tumor with vascular proliferation and four well-known clinical subtypes: classic, endemic, iatrogenic, and HIV-associated [2]. The classic variant is common among men of Mediterranean or European origin aged 50 to 70 years and is exceedingly rare in the Asian population [3]. HIV-seronegative men of African American descent, aged 20 to 30 years, present with an endemic variant of KS. The iatrogenic subtype is seen in immunosuppressed patients, and the epidemic variant is seen in HIV-positive patients [4]. The fifth variant of KS, termed non-epidemic KS, has been recognized in a new subgroup of patients, i.e., HIV-negative men having sex with men with no underlying immunodeficiency. It resembles classic KS in presentation and prognosis [2]. Our patient was a case of classical KS.

The prevalence of HHV-8 infection varies among different populations, with a rate of 1.3% to 4.4% in Southeast Asian and Caribbean regions, 3.7% in healthy individuals, 2.3% in HIV-positive individuals in India, and more than 40% in Africa [4]. HHV-8 is the key causative agent in the development of KS, which explains the high prevalence of KS in countries with a high incidence of HHV-8 infections. It initiates the infection, followed by the proliferation and migration of the infected endothelial cells, leading to tumor formation [5].

KS evolves from early patch to plaque stage into nodules, i.e., tumor stage [6]. All three stages of progression were seen in our patient. KS in a non-AIDS patient is a rare disease. Factors like origin, sex, age, and a patient's immune status affect the incidence of the tumor [7].

The initial or patch stage of KS is characterized by small, thin-walled, bulging, endothelium-lined vessels surrounded by large ectatic vessels and skin appendages, giving rise to the *promontory sign*. Mild inflammation with extravasated erythrocytes, hemosiderin-laden macrophages, and horizontally placed vascular structures can be observed around the lesion, and the papillary dermis is generally intact. The proliferating vascular structures infiltrate the dermis and sometimes subcutis in the plaque stage. The spindle cells concentrate and form bundles around the proliferating vascular channels, and eosinophilic and hyaline globules can be seen. In the classic nodular stage of KS, intersecting, bundled spindle cells and erythrocyte-containing clefts that separate vascular structures are observed. The dilated vascular spaces similar to a cavernous hemangioma may be observed around some lesions. The large cutaneous nodules can ulcerate, and the tumor may exhibit cellular pleomorphism, necrosis, and mitotic figures in this stage [8].

KS lesional cells stain positively with the endothelial markers factor VIII-related antigen, CD31 (platelet endothelial cell adhesion molecule-1, or PECAM-1), and CD34. In the advanced stage of KS, CD34 is more strongly expressed than CD31. D2-40, lymphatic specific marker, vascular endothelial growth factor C receptor-39 (VEGEFR-39), and LYVE-1 (homolog of CD44 glycoprotein receptor for hyaluronan) are expressed by spindle cells of KS. The latency-associated nuclear antigen (HHV8) is the most specific immunohistochemical marker available to help distinguish KS from its mimics [6].

KS is treated with different modalities aimed at achieving disease control, preventing complications, and improving overall survival. The limited disease can be treated with high response rates by surgery, radiotherapy, and intralesional chemotherapy. In classic cases, other localized treatments in the form of intralesional vincristine, imiquimod, interferon alpha-2, and nicotine patches have been used [9].

Systemic treatments like pegylated liposomal doxorubicin (PLD), daunorubicin, bleomycin, vincristine, low-dose interferon alpha, and paclitaxel are recommended for locally aggressive, extensive, and disseminated KS [10]. Most recommendations on the systemic use of drugs are based on retrospective studies in patients with HIV-associated KS.

Paclitaxel therapy is associated with adverse effects such as low blood counts, hair loss, arthralgias, nausea, diarrhea, vomiting, and mouth sores. Our patient developed severe diarrhea after the fourth infusion, which settled on treatment. Etoposide and vinca alkaloids are other treatments recommended for KS with variable efficacy. Various new drugs are currently being evaluated for the treatment of KS, such as intralesional nivolumab for cutaneous KS; nelfinavir for endemic, classic, and HIV-related KS; and pomalidomide with liposomal doxorubicin for refractory KS [5,10].

## Conclusions

KS is an uncommon disease in immunocompetent individuals. This case report demonstrates the importance of recognizing the disease in patients with erythematous to violaceous papulonodular lesions not responding to conventional treatment with steroids or other treatment modalities for common skin diseases, regardless of their immunological status. The role of biopsy and histopathological examination is cardinal for making a diagnosis in these cases so that the treatment can be started at the earliest.
